# An Alternative Approach to Evaluate the Quality of Protein-Based Raw Materials for Dry Pet Food

**DOI:** 10.3390/ani11020458

**Published:** 2021-02-09

**Authors:** Nicolò Montegiove, Roberto Maria Pellegrino, Carla Emiliani, Alessia Pellegrino, Leonardo Leonardi

**Affiliations:** 1 Department of Chemistry, Biology and Biotechnology, Biochemistry and Molecular Biology Section, University of Perugia, Via del Giochetto, 06123 Perugia, Italy; roberto.pellegrino@unipg.it (R.M.P.); carla.emiliani@unipg.it (C.E.); 2 Centro di Eccellenza sui Materiali Innovativi Nanostrutturati (CEMIN), University of Perugia, Via del Giochetto, 06123 Perugia, Italy; 3 Independent Researcher, Via Indipendenza 31/A, 06081 Assisi, Italy; alessiapellegrino@befood-pet.com; 4 Department of Veterinary Medicine, University of Perugia, Via San Costanzo 4, 06126 Perugia, Italy; leonardo.leonardi@unipg.it

**Keywords:** fresh meats, meat meals, soluble protein content, Bradford assay, Kjeldahl method, near-infrared spectroscopy, protein bioavailability, protein profile, amino acid profile, in vitro digestibility

## Abstract

**Simple Summary:**

The protein sources used for the production of dry pet food are mainly made of fresh meats (FMs) and especially meat meals (MMs). The transport and storage conditions of these raw materials, together with thermal and mechanical treatments in the case of MMs, may result in undesirable alterations of food products and their protein content. The aim of this study was to analyze the protein component of the raw materials used for dry pet food production, also testing the use of an alternative method to traditional ones, i.e., the Bradford assay. The results showed that the MMs are lower in quality compared to FMs, both in terms of protein bioavailability and digestibility. Furthermore, the Bradford assay proved to be a quick and simple method to better estimate protein bioavailability in the ingredients used for dry pet food production compared to traditional methods.

**Abstract:**

The majority of dry pet food currently on the market is produced using fresh meats (FMs) and especially meat meals (MMs) as the main protein source. The transport and storage conditions of the raw materials, together with thermal and mechanical treatments in the case of MMs, may result in undesirable alterations of food products and their protein content. This study was conducted to analyze the protein component of three different kinds of raw materials used for dry pet food production, i.e., chicken, pork, and salmon. The quantitative analysis of the protein component was determined using the traditional Kjeldahl method and near-infrared (NIR) spectroscopy, and an alternative method, i.e., the Bradford assay, while the qualitative analysis was performed through SDS-PAGE, followed by Coomassie Blue staining. The amino acid (AA) profile was also evaluated by quadrupole time-of-flight liquid chromatography/mass spectrometry (Q-TOF LC/MS). In addition, the digestibility was tested through in vitro gastric and small intestine digestion simulation. Statistical analysis was performed by the Student’s *t*-test, and data are reported as mean ± SEM, n = 10 (*p* < 0.05). The results showed that the MMs are lower in quality compared to FMs, both in terms of protein bioavailability and digestibility, having a lower soluble protein (SP) content (chicken MM = 8.6 g SP/100 g dry sample; pork MM = 6.2 g SP/100 g dry sample; salmon MM = 7.9 g SP/100 g dry sample) compared to FMs (chicken FM = 14.6 g SP/100 g dry sample; pork FM = 15.1 g SP/100 g dry sample; salmon FM = 13.7 g SP/100 g dry sample). FMs appear, therefore, to be higher-quality ingredients for pet food production. Moreover, the Bradford assay proved to be a quick and simple method to better estimate protein bioavailability in the raw materials used for dry pet food production, thanks to its correlation with the in vitro digestibility.

## 1. Introduction

The European market of dry pet food is constantly expanding, and new formulations are proposed, making it necessary to have a more accurate assessment of the ingredients used in the production process, consisting mainly of meat processing wastes [1].

As far as the protein sources are concerned, most dry pet foods found on the market are produced using two different kinds of raw materials: fresh meats (FMs) and especially meat meals (MMs) [2,3]. Although human grade meats can be found in the pet food market, the majority of FMs are obtained from the meat not fit for human consumption, while MMs derive from animal parts not consumed by humans according to the European Regulation (EC) No 1069/2009 of the European Parliament and of the Council of 21/10/2009. FMs are made up of parts of animals, which are rejected as unfit for human consumption, but which did not show any signs of disease transmissible to humans, while MMs may, in addition, include animal hooves, horns, bristles, and feathers. These MMs are mainly used by pet food manufacturers to increase the amino acid (AA) content of pet kibbles in order to make complete feedstuffs. However, the intensive industrial process that MMs undergo may have a negative impact on their digestibility and cause the onset of oxidation processes and partial degradation of the raw materials [4,5]. Moreover, inappropriate storage conditions could give rise to the proliferation of microorganisms, which degrade the organic component, leading to the development of harmful products, such as biogenic amines [6,7].

Broadly speaking, the transport and storage conditions of the different raw materials, including their packaging and preservation, may result in undesirable alterations of the nutritional and organoleptic characteristics of food; in fact, if the cold chain is not respected, the ingredients may be exposed to physical and microbiological stress (e.g., inappropriate storage temperatures and bacterial proliferation) [8,9].

One of the main causes of chemical changes of pet food is due to the pro-oxidant action of oxygen and light [10,11], capable of inducing the formation of radical species and reactive oxygen species (ROSs) that damage different molecules, proteins included. The products of oxidation caused by ROSs are capable of triggering further oxidative processes at the expense of the protein component of the food [12,13]. The accumulation of protein oxidation products can significantly alter the organoleptic and nutritional properties of food products [14,15]. It is well known that ROSs can either induce direct damages to proteins, oxidizing the AA residues to carbonyl derivatives [16], or first react with carbohydrates and lipids, which form derivatives that, in turn, interact with proteins. Oxidative damage to proteins can, therefore, lead to the formation of radical species on the side chains of AAs, degradation of the peptide bond, or formation of covalent bonds with lipidic or glucidic derivatives [15,17].

The ingredients for product formulations are chosen based on their nutrient content, digestibility, palatability, functionality, availability, and cost. Safe and healthy pet food comes from safe ingredients sourced from well-monitored suppliers; therefore, a better understanding of the ingredients is an important part of pet food production [2,3,6]. A healthy diet should guarantee an adequate intake of all the essential amino acids (EAAs) in order to ensure metabolic functions, normal growth, and maintenance of the animal [18]. Among the EAAs, of particular importance are three AAs: isoleucine, leucine, and valine, also called branched-chain amino acids (BCAAs). These BCAAs are about 35% of the AAs present in the muscle [19], intervene in the synthesis of other important AAs, such as glutamine and alanine, promote protein synthesis, contribute to a stronger immune system, increase muscular endurance, and play a key role in the regulation of energy homeostasis [20,21,22,23]. Another important AA for the pet diet is taurine. This sulfur-containing AA plays a fundamental role in the synthesis of bile acids, regulates the transmission of nerve impulses, and has antioxidant properties [24,25], promoting physical recovery after an intense effort and reducing muscle damage and oxidative stress [26,27]. Moreover, taurine is an EAA for cats that cannot synthesize it and must, therefore, take it with their diet [28,29,30,31].

The choice of raw materials for the production of pet food is thus an essential step for the manufacturing companies as they must provide the necessary nutrients for the development and maintenance of the animal. The ultimate goal of veterinarians, nutritionists, pet food manufacturers, and pet owners is long and healthy lives for dogs and cats, using high-quality ingredients.

The official methods usually used for the protein content analysis of feeds and recognized by the Association of Official Agricultural Chemists (AOAC) are the Kjeldahl method, the Dumas method, and near-infrared (NIR) spectroscopy [32,33,34]. The former is the most commonly used method; however, it is time-consuming, laborious, expensive, and potentially hazardous for the workers because of the required reagents [35,36,37]. NIR spectroscopy is also often used [32,33], as it allows rapid analysis of the protein content; however, this method does not always offer reliable results [38,39,40,41,42]. Given these factors, these two methods can be considered not properly suitable for production processes; furthermore, the Kjeldahl method is found to overestimate the real protein content [43,44,45,46].

At present, the manufacturing companies are lacking in a quick, simple, reproducible, and economical assay that allows the protein content to be quantified in terms of soluble proteins and the protein quality to be evaluated in terms of bioavailability.

Hence, the aim of this work was both to analyze the protein component of the different raw materials used for the production of dry pet food and to investigate a possible alternative to traditional methods for analyzing the protein content of pet food. Chicken, pork, and salmon FMs and MMs were selected to be analyzed in this study as they represent different kinds of animal protein sources, i.e., birds, mammals, and fish, often used in the pet food industry [3].

Protein content analysis was carried out, from both a quantitative and qualitative point of view, in order to evaluate the various raw materials that constitute dry pet food. This may potentially allow establishing new criteria to assess the quality of the raw materials and, therefore, of the final products destined to the production of superior quality dry pet food from the point of view of protein content.

## 2. Materials and Methods

### 2.1. Raw Materials

Raw materials used in this study consisted of: chicken breast meat for human consumption (MHC), 10 batches produced by Italian farms; chicken FM and MM for companion animal food, 10 batches each produced by Italian farms; pork loin MHC, 10 batches produced by Italian farms; pork FM and MM for companion animal food, 10 batches each produced by Italian farms; salmon fillet MHC, 10 batches produced by Norwegian farms; salmon FM and MM for companion animal food, 10 batches of FMs produced by Norwegian farms and 10 batches of MMs produced by Polish farms. All MHCs were purchased in a local supermarket in Perugia, Italy. All FMs and MMs were provided by an Italian pet food manufacturer.

### 2.2. Determination of Moisture Content

Food moisture was calculated according to the official method for the animal feed moisture analysis described by the AOAC [33]. Briefly, an exact amount of raw material (2 g) was evenly distributed on a dish and dried in an oven (Termaks TS 8136, Bergen, Norway) at 135 °C for 2 h. Samples were cooled down at room temperature in a desiccator containing silica gel and weighed using OHAUS Pioneer™ Analytical Balance (OHAUS Corporation, Parsippany, NJ, USA) until a constant and stable weight was reached. Water content was calculated as the difference between the initial and final weight.

### 2.3. Protein Solubilization

Raw FMs or MMs were diluted in a hypotonic solution (10 mM Tris-HCl pH 7.5) at the concentration of 30 g/L (*w*/*v*) and homogenized using ULTRA-TURRAX T25 (IKA^®^-Werke GmbH & Co. KG, Staufen, Germany) for 90 s at 4 °C. After that, 0.1% (*v*/*v*) IGEPAL^®^ CA-630 (Sigma-Aldrich, Saint Louis, MO, USA), a non-denaturing detergent suitable for solubilization of membrane protein complexes, was added to facilitate protein release from the organic matrix. Samples were then sonicated for 30 s at 4 °C using an ultrasonic disintegrator (Soniprep 150, MSE, Heathfield, East Sussex, UK). Finally, a centrifuge at 10,000× *g* for 5 min at 4 °C was carried out to remove the insoluble material. The soluble fraction containing soluble proteins was recovered and used for the Bradford assay and SDS-PAGE coupled with Coomassie Blue staining method.

### 2.4. Determination of Soluble Proteins

Soluble protein content in the samples was determined with the Bradford assay [47] using Quick Start™ Bradford 1× Dye Reagent (Bio-Rad, Hercules, CA, USA) according to the manufacturer’s instructions for one-step determination of protein concentration. The quantitative determination was carried out using the Coomassie Brilliant Blue G-250 dye (Bio-Rad, Hercules, CA, USA), which in the protein-bound form has an absorption peak at 595 nm. The absorbance at 595 nm was measured using a Shimadzu UV-160A UV-Visible Recording Spectrophotometer (Shimadzu Scientific Instruments, Kyoto, Japan). The concentration of the soluble proteins of the samples was obtained from their absorbance using a calibration curve prepared with known concentrations of bovine serum albumin (BSA; Sigma-Aldrich, Saint Louis, MO, USA). Each sample was analyzed in triplicate. Data were expressed as g of soluble protein per 100 g of dry sample.

### 2.5. Determination of Nitrogen Content

The Kjeldahl method was used to assess the sample nitrogen content according to the official method [32,33,34]. Briefly, the same amount of dry raw FMs and MMs (1 g) was digested in sulfuric acid with a catalyst at 420 °C for 1 h to convert the amine nitrogen to ammonium ions. Then, the ammonium ions were converted into ammonia that was separated from the digestion mixture using a distiller UDK 129 (VELP Scientifica, Usmate, MB, Italy). Finally, the ammonia was quantified by titration with a standard solution of hydrochloric acid. Once the total nitrogen of samples was determined, the crude protein content was calculated using the conversion factor of 6.25 to convert the percentage of nitrogen into the percentage of crude protein, since most of the meat proteins typically contain 16% nitrogen [48,49,50]. Each sample was analyzed in duplicate. Data were expressed as g of crude protein per 100 g of dry sample.

### 2.6. Near-Infrared (NIR) Spectroscopy

The determination of the crude protein content via NIR spectroscopy [33,34] was carried out using a TANGO-T FT-NIR Spectrometer (Bruker Optik GmbH, Ettlingen, Germany). The NIR spectra were analyzed using OPUS 7.8 spectroscopy software (Bruker Optik GmbH, Ettlingen, Germany), and the crude protein content was quantified using ready-to-use INGOT^®^ calibration applications (Aunir, Towcester, UK). Four determinations were performed for each sample. Data were expressed as g of crude protein per 100 g of dry sample.

### 2.7. Conversion Factors

In order to correlate the different methods used to estimate the protein content of the raw materials (i.e., Bradford assay, Kjeldahl method, and NIR spectroscopy), appropriate conversion factors were calculated. These factors were obtained for both FMs and MMs as the ratio of the mean values of the protein content measured by the Bradford assay with respect to the official Kjeldahl method, the Bradford assay with respect to the official NIR spectroscopy analysis, and NIR spectroscopy with respect to the Kjeldahl method.

### 2.8. SDS-PAGE and Coomassie Blue Staining Method

The electrophoretic profile of the samples was assessed through SDS-PAGE according to the Laemmli protocol [51]. A quantity of soluble protein extract corresponding to the same amount of each dry sample was mixed with sample buffer 5X (0.5 M Tris-HCl pH 6.8, 10% (*w*/*v*) SDS, 50% (*v*/*v*) glycerol, 0.01% (*w*/*v*) bromophenol blue, and 125 mM dithiothreitol; Sigma-Aldrich, Saint Louis, MO, USA) at a concentration ratio of 4:1 (*v*/*v*). Samples were then boiled for 5 min and electrophoresed on 15% acrylamide gel (Mini-PROTEAN^®^ 3 Cell, Bio-Rad, Hercules, CA, USA) at 40 mA. Gels were later stained with Coomassie Blue R-250 (Bio-Rad, Hercules, CA, USA). Each sample was analyzed in duplicate.

### 2.9. Amino Acid Profile

The amino acid (AA) profile of raw FMs and MMs was assessed after the hydrolysis step using liquid chromatography coupled with mass spectrometry. The hydrolysis of the raw material was carried out according to the method described by Adebiyi et al. [52] with minor modifications. In brief, 2 g of dry powder of each sample was suspended in 15 mL of 0.1 M HCl and homogenized using ULTRA-TURRAX T25 (IKA^®^-Werke GmbH & Co. KG, Staufen, Germany) for 90 s at 4 °C. Then, 50 μL of the suspension was mixed with 150 μL of a freshly prepared solution of 6 N HCl, 3% phenol, and 1% 2-mercaptoethanol and incubated for 30 min at 160 °C in a glass-sealed vial to obtain complete hydrolysis of protein material, as this temperature allows rapid hydrolysis of the protein component with a recovery mean values of the AAs greater than 98%; however, cysteine cannot be detected under these conditions [53,54]. Furthermore, acid hydrolysis can lead to the conversion of glutamine and asparagine into glutamic and aspartic acid [54]. After the hydrolysis step, an aliquot of 50 μL of the hydrolyzed mixture was diluted with distilled water and filtered through C18 solid-phase extraction (SPE) cartridge for the defatting step. The ion-pairing chromatography (IPC) method was used to achieve a wide separation of AAs with 150 × 2.1 mm, 3 μm ACME^TM^ Amide C18 column (Phase Analytical Technology LLC, State College, PA, USA) thermostated at 50 °C. The separation of AAs was achieved using a flow of 0.35 mL/min of a binary gradient of 0.3% heptafluorobutyric acid in water (solvent A) and 0.1% formic acid in methanol (solvent B). The initial condition was 2% of B for 2 min, followed by a gradient from 2 to 80% of B in 5 min, and a final isocratic step of 8 min.

The spectrometer was operated in high-resolution full scan mode, monitoring positive ions. The quantitative data were obtained by external calibration in the range 0.05–2.5 μg/mL of a homemade mix of AA in pure methanol. In the end, 1 μL of the sample was loaded into Agilent 6530 Q-TOF LC/MS (Agilent Technologies, Inc., Santa Clara, CA, USA) for AA profile analysis. Each sample was analyzed in duplicate. Data were expressed as g of AA per 100 g of dry sample.

### 2.10. In Vitro Digestibility

In vitro digestibility was assessed according to Vervaeke et al. [55] with few modifications.

Gastric digestion simulation: samples were finely ground (<1 mm particle size). A quantity corresponding to 500 mg of dry matter was weighted for each sample, taking into account the moisture content previously measured. Each sample was incubated with 20 mL of a Pepsin-HCl solution (0.075 N HCl, pepsin from porcine gastric mucosa 2 g/L; Sigma-Aldrich, Saint Louis, MO, USA) in a 50 mL tube in a shaking water bath at 39 °C for 2 h.

Small intestine digestion simulation: first, the pH level was adjusted to 7.5 with 1 N NaOH. Then, 20 mL of 10 g/L pancreatin from porcine pancreas, 1.6 g/L trypsin from porcine pancreas, 3.1 g/L α-chymotrypsin from bovine pancreas, 1.3 g/L protease from *Streptomyces griseus*, and 1 g/L lipase from *Rhizopus oryzae* (Sigma-Aldrich, Saint Louis, MO, USA) dissolved in phosphate-buffered solution pH 7.5 (3.92 g NaHCO_3_, 3.72 g Na_2_HPO_4_, 0.23 g KCl, 0.19 g NaCl, 0.12 g MgCl_2_, and 0.08 g CaCl_2_ in 1 L of distilled water; [56]) were added to each tube. Immediately prior to the addition of the enzymatic solution, bile salts (50% cholic acid sodium salt and 50% deoxycholic acid sodium salt; Sigma-Aldrich, Saint Louis, MO, USA) were added to each tube at a final concentration of 25 g/L. Finally, the tubes were placed in a shaking water bath at 39 °C for 4 h.

Collection of the undigested fraction: after enzymatic digestion, the preparation was centrifuged (3000× *g* for 10 min at 4 °C), washed twice with distilled water, and re-centrifuged (3000× *g* for 5 min at 4 °C). The undigested residue was dried at 135 °C until constant weight.

In order to determine the dry matter digestibility of the samples, the residue obtained from each tube after the in vitro digestion was weighed, and the digestibility was calculated with the following equation:

In vitro digestibility % = [100 − [(dry residue weight × 100)/dry sample weight]

### 2.11. Statistical Analysis

Data shown in this study, regarding the analysis of the protein content of the raw materials used for the production of dry pet food, are reported as mean values of the ten analyzed batches ± standard error of the mean (SEM). The Student’s *t*-test was used to analyze the significance of the protein content differences between the means of each type of FM and its relative MM (chicken FM vs. chicken MM; pork FM vs. pork MM; salmon FM vs. salmon MM) in each of the three methodologies considered in this study (i.e., Bradford assay, Kjeldahl method, and NIR spectroscopy). The Student’s *t*-test was also used to evaluate the significance of the differences in the AA content assessed by acid hydrolysis, followed by Q-TOF LC/MS analysis and the significance of the differences in the in vitro digestibility between chicken, pork, and salmon FMs and their relative MMs. The level of significance for the data was set at *p* < 0.05. Pearson’s correlation analysis was used to highlight the possible relation between the FM and MM protein content obtained with two different methods (Bradford assay vs. Kjeldahl method; Bradford assay vs. NIR spectroscopy; NIR spectroscopy vs. Kjeldahl method) and between the raw material in vitro digestibility and the protein content assessed by the different methods. All statistical tests were done using GraphPad Prism 6.00 for Windows (GraphPad Software, San Diego, CA, USA).

## 3. Results

### 3.1. Protein Content

**Figure 1:**
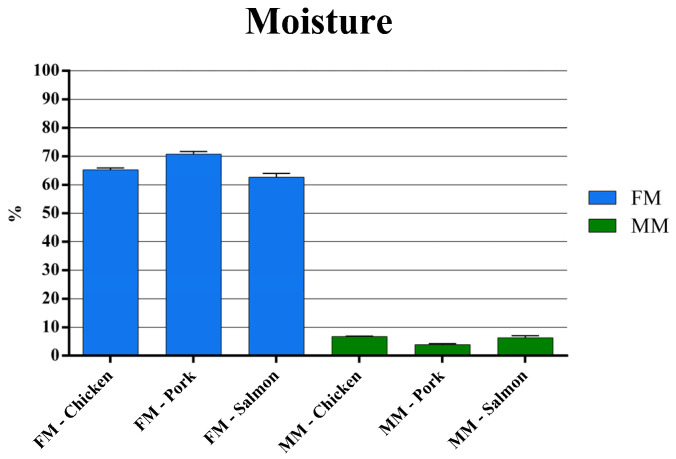
Water content in chicken, pork, and salmon fresh meat (FM) and meat meal (MM). Data are reported as mean ± SEM, n = 10.

Prior to the assessment of the soluble protein content by the Bradford assay, the moisture level was evaluated for each raw material. The results shown in Figure 1 reveal how FMs exhibit higher water content compared to MMs. The humidity level in FMs ranges from about 60% in the case of salmon to about 70% in the case of pork, whereas a water content lower than 10% is peculiar to all MMs.

**Figure 2:**
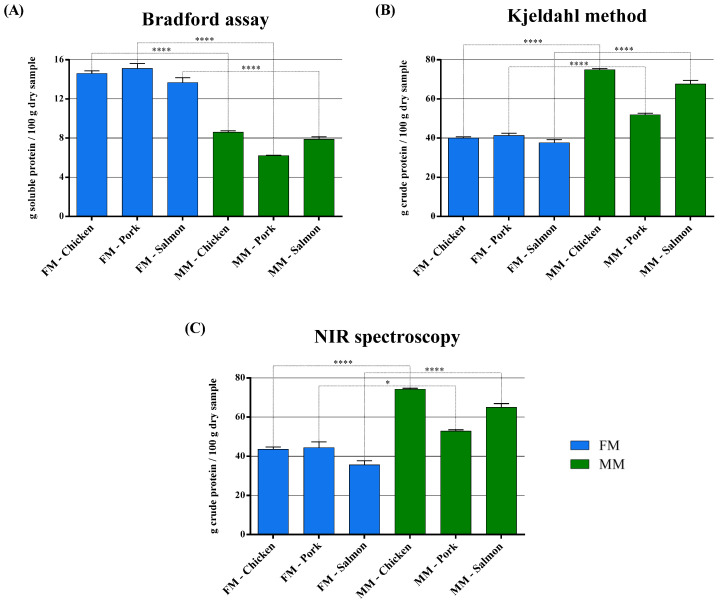
(**A**) The soluble protein content of chicken, pork, and salmon FM and MM determined by the Bradford assay. (**B**) The crude protein content of chicken, pork, and salmon FM and MM determined by the Kjeldahl method. (**C**) The crude protein content of chicken, pork, and salmon FM and MM determined by NIR spectroscopy. Data are reported as mean ± SEM, n = 10. * *p* < 0.05, **** *p* < 0.0001.

Subsequently, the Bradford assay was performed on chicken, pork, and salmon FMs or MMs for companion animal food, taking into account the different water content (Figure 1), and it has been found that FMs contain a higher level of soluble proteins as compared to MMs (Figure 2A and Table 1). Chicken FM has a content of about 1.7 times higher in soluble proteins (14.6 g/100 g of dry sample) compared to MM, as well as salmon FM (13.7 g/100 g of dry sample), while pork is the one that has the highest concentration of soluble proteins (15.1 g/100 g of dry sample) compared to the relative MM, of about 2.4 times.

The official Kjeldahl method instead shows a higher crude protein content of MMs compared to FMs for companion animal food (Figure 2B and Table 1). Chicken MM has a crude protein content of about 1.9 times higher compared to FM, similar to what is observed for salmon MM where the content is about 1.8 times higher; while in pork, the content of crude proteins is only about 1.3 times higher. Moreover, it is in chicken MM where the highest concentrations of crude proteins are found, about 74.9 g/100 g of dry sample, followed by salmon and pork.

Protein concentration was also assessed by another official method, i.e., NIR spectroscopy (Figure 2C and Table 1), and even in this case, MMs show a higher crude protein concentration compared to FMs. Salmon MM has a content of about 1.8 times higher in crude proteins compared to FM, chicken MM ranges about 1.7 times higher, while in pork, the content of crude proteins is only about 1.2 times higher compared to the relative FM. Additionally, here, like in the Kjeldahl analysis, it is in chicken MM where the highest concentrations of crude proteins are found, about 74.2 g/100 g of dry sample, followed by salmon and pork.

**Figure 3:**
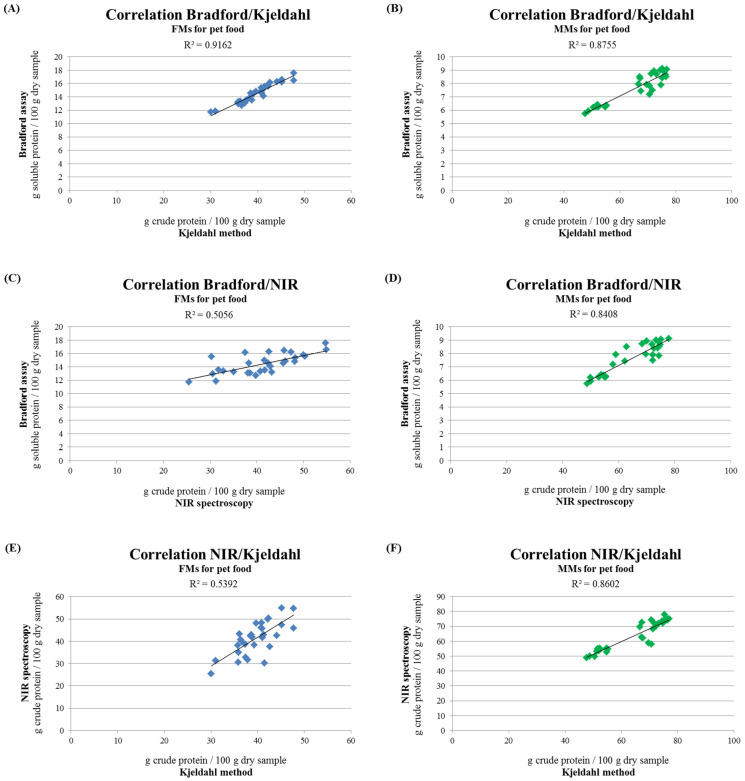
(**A**,**B**) Correlation between the soluble and crude protein content of chicken, pork, and salmon FM and MM determined by the Bradford assay and the Kjeldahl method. (**C**,**D**) Correlation between the soluble and crude protein content of chicken, pork, and salmon FM and MM determined by the Bradford assay and NIR spectroscopy. (**E**,**F**) Correlation between the crude protein content of chicken, pork, and salmon FM and MM determined by NIR spectroscopy and the Kjeldahl method. Data are reported as mean, n = 10.

The data concerning the analysis of the protein content of raw materials with the different methods showed different trends (Figure 2 and Table 1). However, comparing the data obtained, the presence of a direct correlation between the Bradford assay and the Kjeldahl method comes to light, both for FMs (R^2^ = 0.9162) (Figure 3A) and MMs (R^2^ = 0.8755) (Figure 3B). The correlation is strictly dependent on the nature of the raw materials; in fact, average factors of 0.36 and 0.12 are found between the values obtained by the Bradford assay and the Kjeldahl method for FMs and MMs, respectively.

On the contrary, no strong correlations are found neither between the Bradford assay and NIR spectroscopy (R^2^ = 0.5056) nor between NIR spectroscopy and the Kjeldahl method (R^2^ = 0.5392) for all the FMs analyzed (Figure 3C,E), while a better correlation has been found for MMs (Figure 3D,F) R^2^ = 0.8408 and R^2^ = 0.8602, respectively. The ratio between the Bradford assay and NIR spectroscopy, as far as MMs are concerned, corresponds to a factor of 0.12, while for the NIR/Kjeldahl comparison, the ratio is 0.99.

### 3.2. Protein Profile

**Figure 4:**
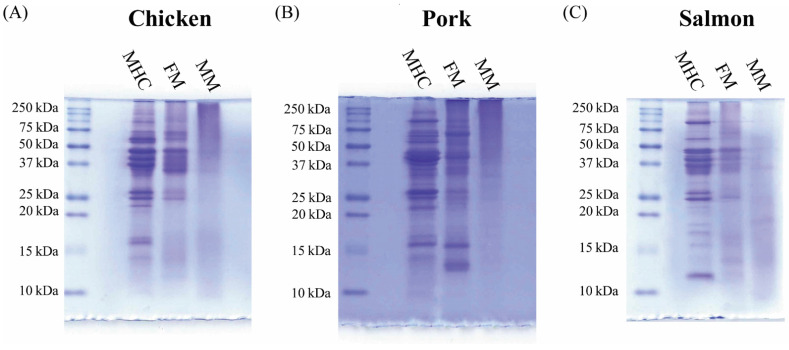
Protein profile of meat for human consumption (MHC), FM, and MM of chicken (**A**), pork (**B**), and salmon (**C**) evaluated by SDS-PAGE, followed by Coomassie Blue staining method.

The electrophoretic profile of soluble proteins extracted from FMs and MMs for companion animal food with those obtained from meats for human consumption (MHCs) was compared. The results shown in Figure 4 were chosen as representative of the 10 batches analyzed. The results show that the MHC and FM protein profiles are mostly overlapping, while MMs display a substantially different pattern, as evidenced by the presence of smear on the gel instead of net bands. Chicken and pork FMs are the ones showing the most marked protein banding patterns.

### 3.3. Amino Acid Profile

All the AAs detected are shown in Table 2. The results indicate that MMs have a higher total AA content than FMs, for all the raw materials analyzed; however, the AA composition is found to be different. The data highlight that MMs are significantly enriched in two AAs: glycine and proline, whose overall concentration ranges from about 22–24% of the total AA composition in the case of pork and salmon MMs to about 29% in the case of chicken MMs. The glycine content results to be 4.7 times higher in chicken MMs compared to FMs, being 8.0 g per 100 g of dry sample. The same trend is observed for salmon MMs, where the content is 2.7 times higher (4.6 g/100 g of dry sample), while in the case of pork, the difference between MMs and FMs is less pronounced. As far as the proline content is concerned, results show that this AA is 5.6 times higher in chicken MMs compared to FMs, resulting to be 5.0 g per 100 g of dry sample. The same trend is observed for pork and salmon MMs, where the proline content is 4.5 g per 100 g of dry sample and 4.8 g per 100 g of dry sample, respectively. These values are 2.4 and 5.7 times higher than the corresponding FMs. The table also shows that other highly represented AAs are isoleucine, leucine, phenylalanine, and valine, both in MMs and FMs, whose overall concentration ranges from 68% of the total AA composition for pork FMs to 76–77% in the case of salmon and chicken FMs. In addition, the analysis also reveals that the arginine content is significantly higher in MMs than in FMs, especially in chicken MMs (331 mg/100 g of dry sample). On the contrary, pork and salmon FMs compared to MMs are significantly richer in taurine. This is especially the case for pork FMs, where the taurine content is 5.8 times higher (99 mg/100 g of dry sample) than the relative MMs. However, the highest taurine concentration (170 mg/100 g of dry sample) is found in salmon FMs.

### 3.4. In Vitro Digestibility

**Figure 5:**
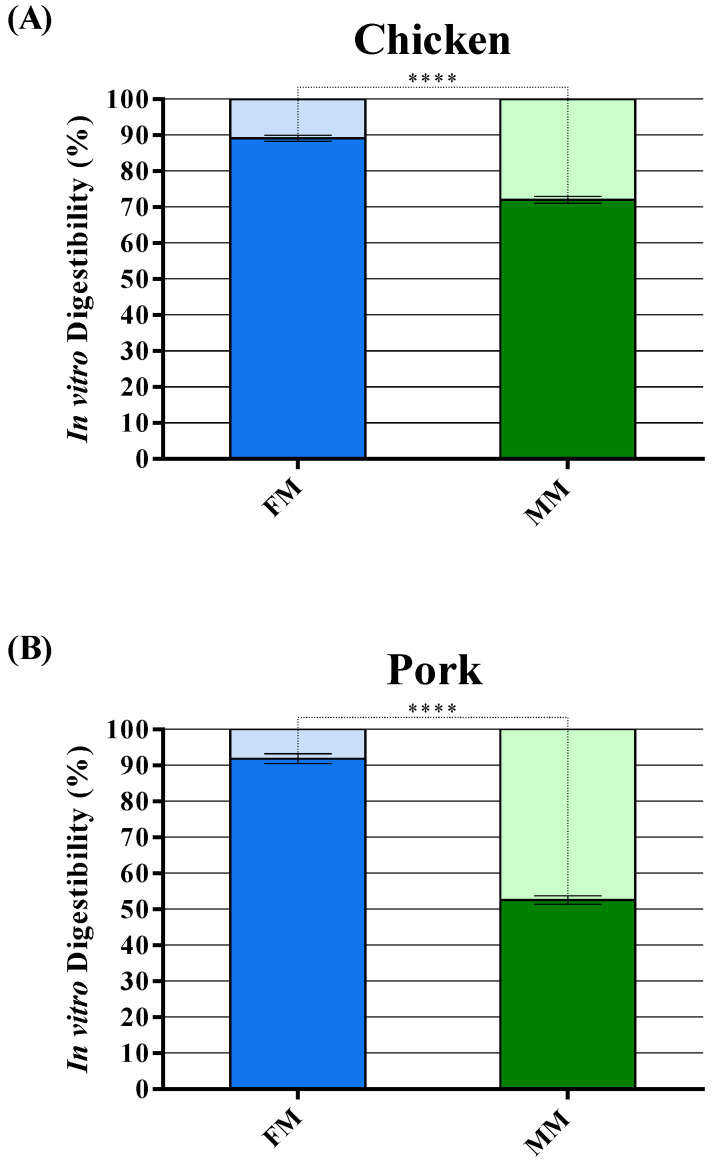

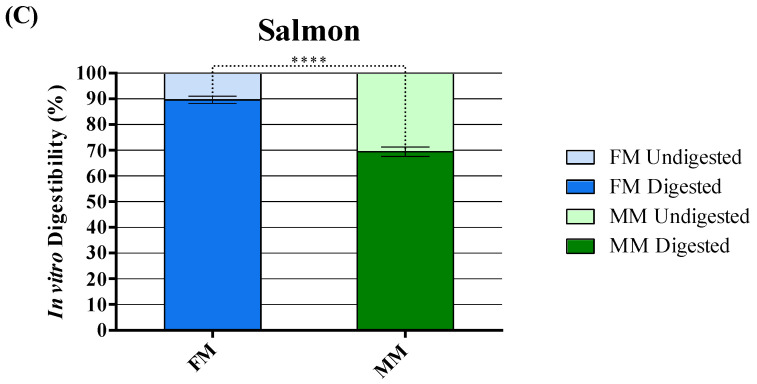
Digestibility of chicken (**A**), pork (**B**), and salmon (**C**) FM and MM analyzed with in vitro digestibility assay. Data are reported as mean ± SEM, n = 10. **** *p* < 0.0001.

The in vitro digestibility of both FMs and MMs for companion animal food was analyzed by a gastric and small intestine in vitro digestion simulation. At the end of the reaction, the undigested insoluble material was dried and weighed to have an estimate of the in vitro digestibility on a dry matter basis. FMs show a range of digestibility varying between about 89 and 92%, while only 52–72% of MMs are completely digested (Figure 5A–C).

**Figure 6:**
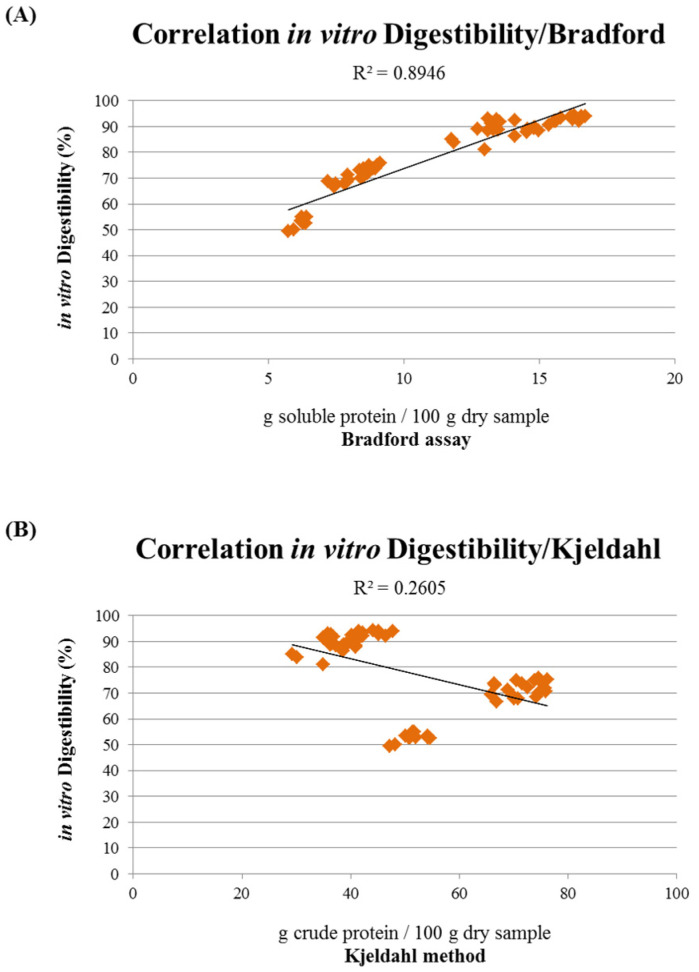

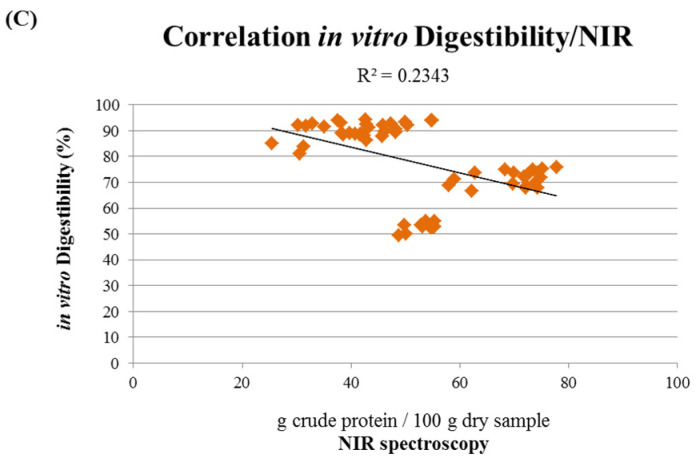
(**A**) Correlation between in vitro digestibility assay and soluble protein content evaluated by the Bradford assay of chicken, pork, and salmon FM and MM. (**B**) Correlation between in vitro digestibility assay and crude protein content evaluated by the Kjeldahl method of chicken, pork, and salmon FM and MM. (**C**) Correlation between in vitro digestibility assay and crude protein content evaluated by NIR spectroscopy of chicken, pork, and salmon FM and MM. Data are reported as mean ± SEM, n = 10.

The obtained values of digestibility were then correlated with the values of the Bradford assay, highlighting a linear correlation (R^2^ = 0.8946) between the soluble protein content and the digestibility of the raw materials used for the production of dry pet food (Figure 6A). The digestibility of the different ingredients was also compared with the Kjeldahl method and NIR spectroscopy; however, the results show how there is a weak inverse correlation between the crude protein content and the digestibility (Figure 6B,C).

## 4. Discussion

The nutrient content of the raw materials needed for pet food production, together with their digestibility, palatability, functionality, availability, and cost, are all key factors in driving the ingredient choice. Most of the pet foods on the European market today are produced using MMs or FMs as protein sources, which could have different degrees of nutritional quality, influencing the quality of the final products.

In order to evaluate the protein content of raw materials used for the production of dry pet food, we first performed the Bradford assay on chicken, pork, and salmon FMs or MMs for companion animal food, taking into account the different water content (Figure 1); however, only 10 batches of each ingredient were available for testing in this study. The results showed that FMs contained a higher level of soluble proteins as compared to MMs (Figure 2A and Table 1), and this happened for all the raw materials analyzed. Then, the Kjeldahl method was performed to compare the results of the proposed alternative method with the official one. The Kjeldahl method showed a higher crude protein content of MMs compared to FMs for companion animal food (Figure 2B and Table 1). This could be due to the different solubility of the proteins in the different samples. In fact, MMs may contain hooves, horns, bristles, and feathers, which contain a big amount of keratin, collagen, and elastin, notably insoluble or poorly soluble proteins. These fibrous proteins tend to be insoluble, tough, and resistant to digestion compared to the globular proteins, which instead tend to be fairly soluble and have relatively high digestibility characteristics [44,57].

Protein concentration was also evaluated by NIR spectroscopy (Figure 2C and Table 1), another official method used for the analysis of the pet food crude protein content [33,34]. In this case, MMs showed a higher crude protein concentration compared to FMs, revealing a situation similar to that found with the Kjeldahl method. This could be justified by the fact that both the Kjeldahl method and NIR spectroscopy evaluate the total protein content of the sample, making no distinction between soluble and insoluble ones [32,33,34,58].

The observed correlation between the Kjeldahl method and Bradford assay, both for FMs and MMs (Figure 3A,B), allowed to calculate factors, which correlate the two methods. This trend of close dependence between the unofficial, i.e., the Bradford assay, and the official method, i.e., the Kjeldahl method, for the analysis of the protein content of pet food raw materials, could be then useful for the pet food industry. In fact, it could have a dual purpose, on the one hand, to estimate the effective soluble protein content and, on the other hand, the fact of being able to have an estimate of the total proteins using a faster and less dangerous method compared to the traditional and officially used method. These factors, in fact, could be initially obtained and calculated by the pet food manufacturers, comparing the official methods, e.g., the Kjeldahl method, with the unofficial Bradford assay for each different ingredient, and then they could be used as conversion factors for a quick analysis of the raw material protein content.

On the contrary, for all the FMs analyzed, no strong correlation was found between NIR spectroscopy and the other methods (Figure 3C,E), thus preventing the possibility of electing NIR spectroscopy as a valid technique for estimating the protein content for each type of raw material. This could be due to the difficulty of NIR spectroscopy in making a correct evaluation in wet samples, given that FMs have a high level of moisture. This is in agreement with previous studies in the literature, which show that NIR spectroscopy provides a better estimate of the chemical composition, including proteins, in dried samples than in raw meat, by virtue of their more homogenous physical appearance and reduced water content [39,40,41]. Furthermore, temperature affects the energy absorbed by water, reducing the accuracy of the NIR analysis on wet samples [39,42].

We, therefore, propose that, while the official Kjeldahl method could be used for crude protein content determination, the Bradford assay could be preferred when the assessment of soluble protein needs to be achieved. However, the direct correlation between the two methods could allow the Bradford assay, a quick and simple technique [44,47], to be used to also have an estimate of the total proteins after obtaining the right conversion factor. The time required for all the sample preparation and the analysis through the Bradford assay is approximately 15 min, while the Kjeldahl method takes about 1 h and a half to be performed starting from the raw material. The NIR spectroscopy analysis is indeed very quick, less than a minute, but the time required for sample preparation depends on the material; however, as stated before, NIR spectroscopy is not completely reliable when it comes to wet samples.

Subsequently, in order to evaluate the quality of protein profile in the samples, we compared the electrophoretic profile of soluble protein extracted from FMs and MMs used for dry pet food production with those obtained from MHCs. As shown in Figure 4, the MHC and FM profiles were mostly overlapping, while MMs displayed a substantially different pattern, suggesting a potential partial degradation of proteins, as evidenced by the presence of smear on the gel instead of net bands [59]. This is in agreement with the fact that MMs are produced through an intensive process that may determine a partial degradation of the raw material [4,5,6,7,8,9]. The absence of smear, which would account for smaller peptides, in the lower part of the gel of MM samples, suggested that their dimensions were so small (smaller than 10 kDa) that they had not been retained during the electrophoretic run. The small peptides that are formed could still be useful in the feeding of pets as they could be hypoallergenic and more digestible [60]. However, chicken and pork FMs were the ingredients, showing the most marked protein banding patterns among those tested; this finding is in line with the evidence showing that these two types of FMs are those that usually have a high protein content [61,62,63].

The AA composition of both FMs and MMs used for dry pet food production was then analyzed by Q-TOF LC/MS. Results shown in Table 2 indicated that the highest concentrations were found for glycine, isoleucine, leucine, phenylalanine, proline, and valine. Both FMs and MMs are rich in some EAAs, namely phenylalanine and the three BCAAs, especially for FMs, where they represent more than two-thirds of the total AA content. BCAAs contribute to muscle growth, muscle glucose uptake, and insulin resistance [19,20], while phenylalanine is a fundamental constituent of proteins and plays a role as a precursor of catecholamines (e.g., L-DOPA, dopamine, noradrenaline, and adrenaline) [64]. Conversely, MMs showed a higher level of glycine and proline, the most abundant AAs in collagen [65,66,67], suggesting the presence of this protein in the MMs analyzed. These findings are consistent with the MM composition, which, according to the European Regulation, can contain animal parts rich in fibrous proteins (e.g., keratin, collagen, and elastin), which tend to be less soluble and digestible [44,57].

In addition, the arginine content was found to be higher in MMs, especially in chicken MMs (Table 2). This could be important for cats as they have a higher requirement of this AA compared to dogs [68,69,70]. On the contrary, in the case of pork and salmon, FMs were found to be richer in taurine compared to MMs, with the highest concentration measured in salmon (Table 2). In fact, it has been shown that salmon FM has higher taurine content than other fish meats used in the preparation of pet food [28]. This finding is interesting since taurine is an extremely important EAA for cats [28,29,30,31].

These data, therefore, demonstrate how FMs could be a better choice compared to MMs as pet food raw materials from a protein point of view, allowing the pet food industries to have alternative ingredients that could be more appealing for the production of premium and super-premium pet foods.

Finally, the in vitro digestibility of both FMs and MMs for companion animal food was analyzed (Figure 5A–C). Results showed how FMs had a higher in vitro digestibility than MMs. These findings are consistent with the hypothesis that MMs are enriched in insoluble or poorly soluble proteins that are less bioavailable for digestion and absorption, such as collagen, resulting in a lower nutritional value compared to FMs [44,57]. In fact, an in vivo study showed how rendered poultry by-product meals are more difficult to digest than meats in the small intestinal tract, probably due to the presence of underprocessed feathers that seem to have a negative influence that results in a decrease of the in vivo digestibility [71].

It is thus clear that a method able to discriminate the soluble protein content of raw materials could be useful for predicting the digestibility and bioavailability of the protein content of the final products. In this context, the Bradford assay could be an easy and quick method that can, on the one hand, evaluate the soluble protein content of the ingredients and, on the other hand, allow a prediction of the digestibility index of the pet food raw materials. In fact, by correlating the Bradford assay values with the relative in vitro digestibility of the different raw materials, a linear correlation emerged (Figure 6A), suggesting its predictive potential; however, in vivo studies are necessary to confirm these results. On the contrary, both the Kjeldahl method and NIR spectroscopy did not show any significant correlation with the in vitro digestibility, whereas they seemed to have an inverse correlation (Figure 6B,C). This is probably due to the fact that these methods are less suitable for assessing the content of soluble and more digestible proteins [44,57], while the Bradford assay seems to disclose a good correlation with digestibility.

## 5. Conclusions

This study has shown how different raw materials display a distinct quantitative and qualitative protein composition and diverse degrees of digestibility. We suggest the evaluation of the soluble protein content in raw materials by the Bradford assay, whose protein values also show a good correlation with the in vitro digestibility.

This investigation highlights how FMs, compared to MMs, have higher soluble protein content, as shown by the Bradford assay and by the analysis of the protein profile. Besides, a better digestibility of the protein content in FMs is demonstrated by in vitro gastric and small intestine digestion simulation, which is directly proportional to the amount of soluble and, therefore, more bioavailable and digestible proteins. Moreover, the quantitative analysis of the AA profile exhibits how MMs are richer in glycine and proline, which indicate higher proportions of less digestible fibrous proteins.

This study also emphasizes how the Bradford assay could be an alternative to the official Kjeldahl method, as it shows a high linear correlation with the official method in the estimate of the protein content. This could be useful for manufacturing companies that could easily produce conversion factors for each type of raw material that can thus be used to estimate the total protein content and, at the same time, have better estimates of the portion of the protein that would have a higher value for the animal. On the other hand, the NIR spectroscopy analysis has not been found to be equally effective in evaluating the protein content in FMs.

Further in vivo studies will be necessary to deepen and confirm these preliminary results. This could also allow drawing a clearer idea of the relative digestibility indexes of the different raw materials used for dry pet food production.

## Figures and Tables

**Table 1:** Soluble protein (SP) and crude protein (CP) content of chicken, pork, and salmon FM and MM determined by Bradford assay, Kjeldahl method, and NIR spectroscopy. Data are reported as mean ± SEM, n = 10.

Raw Material	FM			MM		
	Bradford (g SP/100 g Dry Sample)	Kjeldahl (g CP/100 g Dry Sample)	NIR (g CP/100 g Dry Sample)	Bradford (g SP/100 g Dry Sample)	Kjeldahl (g CP/100 g Dry Sample)	NIR (g CP/100 g Dry Sample)
**Chicken**	14.6 ± 0.3	40.0 ± 0.7	44 ± 1	8.6 ± 0.1	74.9 ± 0.6	74.2 ± 0.5
**Pork**	15.1 ± 0.5	41 ± 1	44 ± 3	6.20 ± 0.06	51.9 ± 0.8	52.8 ± 0.8
**Salmon**	13.7 ± 0.5	38 ± 2	36 ± 2	7.9 ± 0.3	68 ± 2	65 ± 2

FM = fresh meat, MM = meat meal.

**Table 2:** Amino acid (AA) content of chicken, pork, and salmon FM and MM evaluated by Q-TOF LC/MS analysis. Data are reported as mean ± SEM, n = 10.

AA Content (g AA/100 g Dry Sample)	Type of Sample								
	Chicken			Pork			Salmon		
	FM	MM	*p*-Value	FM	MM	*p*-Value	FM	MM	*p*-Value
**Alanine**	0.41 ± 0.03	0.8 ± 0.1	**	0.44 ± 0.05	0.75 ± 0.08	**	0.28 ± 0.03	0.8 ± 0.1	**
**Arginine**	0.040 ± 0.008	0.33 ± 0.02	****	0.06 ± 0.01	0.113 ± 0.007	**	0.032 ± 0.005	0.195 ± 0.007	****
**Aspartic acid**	0.010 ± 0.001	ND	****	0.0090 ± 0.0007	0.0070 ± 0.0006	*	ND	0.017 ± 0.002	****
**Glutamic acid**	0.40 ± 0.05	2.8 ± 0.3	****	0.50 ± 0.08	1.4 ± 0.2	***	0.30 ± 0.03	1.6 ± 0.2	***
**Glutamine**	ND	0.056 ± 0.002	****	0.0092 ± 0.0005	0.042 ± 0.007	**	ND	0.0260 ± 0.0009	****
**Glycine**	1.7 ± 0.1	8.0 ± 0.4	****	1.7 ± 0.1	2.1 ± 0.1	*	1.7 ± 0.1	4.6 ± 0.4	****
**Histidine**	0.0232 ± 0.0008	0.04 ± 0.01	ns	0.047 ± 0.006	ND	****	0.0173 ± 0.0007	0.0046 ± 0.0004	****
**Isoleucine**	6.9 ± 0.2	7.7 ± 0.3	ns	5.0 ± 0.2	5.4 ± 0.1	ns	6.2 ± 0.4	7.6 ± 0.5	ns
**Leucine**	5.3 ± 0.2	7.8 ± 0.3	****	5.4 ± 0.2	6.6 ± 0.2	***	5.1 ± 0.2	8.0 ± 0.2	****
**Lysine**	0.9 ± 0.1	1.6 ± 0.1	**	1.2 ± 0.2	0.74 ± 0.05	*	0.9 ± 0.1	1.1 ± 0.2	ns
**Methionine**	0.40 ± 0.09	0.6 ± 0.2	ns	0.7 ± 0.1	1.23 ± 0.08	**	0.5 ± 0.1	1.8 ± 0.3	***
**Phenylalanine**	2.4 ± 0.2	4.3 ± 0.5	**	3.1 ± 0.1	3.6 ± 0.2	ns	2.4 ± 0.1	3.9 ± 0.5	*
**Proline**	0.88 ± 0.07	5.0 ± 0.3	****	1.85 ± 0.07	4.5 ± 0.2	****	0.84 ± 0.09	4.8 ± 0.4	****
**Serine**	0.035 ± 0.004	0.32 ± 0.01	****	0.218 ± 0.007	ND	****	0.044 ± 0.004	0.075 ± 0.005	***
**Taurine**	0.10 ± 0.02	0.14 ± 0.01	ns	0.099 ± 0.007	0.017 ± 0.001	****	0.170 ± 0.008	0.11 ± 0.01	****
**Threonine**	0.08 ± 0.01	0.10 ± 0.01	ns	0.088 ± 0.009	0.065 ± 0.004	*	0.060 ± 0.008	0.08 ± 0.02	ns
**Tryptophan**	0.09 ± 0.01	0.060 ± 0.009	*	0.065 ± 0.009	0.033 ± 0.001	**	0.07 ± 0.01	0.06 ± 0.02	ns
**Tyrosine**	0.40 ± 0.07	0.8 ± 0.1	*	0.70 ± 0.08	1.06 ± 0.09	**	0.43 ± 0.08	0.6 ± 0.1	ns
**Valine**	3.4 ± 0.3	4.6 ± 0.3	**	3.02 ± 0.06	2.84 ± 0.05	*	3.0 ± 0.3	3.9 ± 0.3	ns

FM = fresh meat, MM = meat meal. ND = not detected. ns = difference is not statistically significant, * *p* < 0.05, ** *p* < 0.01, *** *p* < 0.001, **** *p* < 0.0001.

## Data Availability

Not applicable.
